# A PHF8 Homolog in *C*. *elegans* Promotes DNA Repair via Homologous Recombination

**DOI:** 10.1371/journal.pone.0123865

**Published:** 2015-04-08

**Authors:** Changrim Lee, Seokbong Hong, Min Hye Lee, Hyeon-Sook Koo

**Affiliations:** Department of Biochemistry, College of Life Science & Biotechnology, Yonsei University, Seoul, Republic of Korea; The University of Hong Kong, HONG KONG

## Abstract

PHF8 is a JmjC domain-containing histone demethylase, defects in which are associated with X-linked mental retardation. In this study, we examined the roles of two PHF8 homologs, JMJD-1.1 and JMJD-1.2, in the model organism *C*. *elegans* in response to DNA damage. A deletion mutation in either of the genes led to hypersensitivity to interstrand DNA crosslinks (ICLs), while only mutation of *jmjd-1*.*1* resulted in hypersensitivity to double-strand DNA breaks (DSBs). In response to ICLs, JMJD-1.1 did not affect the focus formation of FCD-2, a homolog of FANCD2, a key protein in the Fanconi anemia pathway. However, the dynamic behavior of RPA-1 and RAD-51 was affected by the mutation: the accumulations of both proteins at ICLs appeared normal, but their subsequent disappearance was retarded, suggesting that later steps of homologous recombination were defective. Similar changes in the dynamic behavior of RPA-1 and RAD-51 were seen in response to DSBs, supporting a role of JMJD-1.1 in homologous recombination. Such a role was also supported by our finding that the hypersensitivity of *jmjd-1*.*1* worms to ICLs was rescued by knockdown of *lig-4*, a homolog of Ligase 4 active in nonhomologous end-joining. The hypersensitivity of *jmjd-1*.*1* worms to ICLs was increased by *rad-54* knockdown, suggesting that JMJD-1.1 acts in parallel with RAD-54 in modulating chromatin structure. Indeed, the level of histone H3 Lys9 tri-methylation, a marker of heterochromatin, was higher in *jmjd-1*.*1* cells than in wild-type cells. We conclude that the histone demethylase JMJD-1.1 influences homologous recombination either by relaxing heterochromatin structure or by indirectly regulating the expression of multiple genes affecting DNA repair.

## Introduction

Histones H3 and H4 are methylated on several amino acid residues, and this methylation can proceed up to the triple level at individual amino acids, usually lysines or less frequently arginines. Histone methylation patterns differ greatly between active and inactive genes, suggesting that histone methylation has a great impact on chromatin structure [1]. A well-known example is the histone H3 Lys9 tri-methylation and Lys4 hypomethylation in heterochromatin [2]. DNA damage signaling as well as gene expression, is influenced by histone methylation, as in the case of 53BP1 binding to chromatin containing methylated H3K79 or H4K20 [3,4]. The DNA damage checkpoint protein MDC1 is demethylated in response to double-strand DNA breaks, and this allows its ubiquitination [5]. The methylation of the tumor suppressor p53 promotes its association with 53BP1 leading to transcriptional activation and apoptosis [6].

Histones methylated on lysines are demethylated by two classes of enzymes, LSD and JmjC demethylases, with very different reaction mechanisms [1,7,8]. The LSD (lysine-specific demethylase) family has only two members in mammals and removes methyl groups by oxidizing amines using FAD and oxygen. The other family, JmjC (with Jumonji C domains), has more than twenty members, and requires Fe^2+^ and α-ketoglutarate for catalysis. A JmjC demethylase, PHF8, targets histone H3 mono- and di-methyl Lys9 (H3K9me1 and H3K9me2), and its mutation is associated with X-linked mental retardation (XLMR) [9]. In the model organism *C*. *elegans*, there are two close homologs of PHF8, JMJD-1.1 and JMJD-1.2, encoded by F43G6.6 and F29B9.2 open reading frames, respectively. Interestingly, JMJD-1.2 is expressed in neurons and is involved in the movement of worms, which agrees with the fact that its human homolog PHF8 is associated with XLMR. JMJD-1.2 targets H3K9me2 like PHF8, as well as H3 di-methyl Lys27 (H3K27me2) [9], and induces the expression of target genes by binding to H3K4me3 via a PHD domain [10].

In this work, we investigated whether and how the two PHF8 homologs in *C*. *elegans*, JMJD-1.1 and JMJD-1.2, affect worm survival in response to DNA damage. JMJD-1.2 demethylates H3K9me2 and H3K27me2, and its closest homolog JMJD-1.1 is likely to have a similar specificity for particular histone residues. It is known that the di- and tri-methylation of H3K9 and H3K27 have been associated with inactive genes and heterochromatin [1]. Therefore, it seemed very likely that the two PHF8 homologs influence responses to DNA damage by modulating chromatin structure. Indeed, we show here that the two proteins, especially JMJD-1.1, influence DNA repair in response to interstrand DNA crosslinks and double-strand DNA breaks.

## Results

### JMJD-1.1 is required for resistance to interstrand DNA crosslinks and double-strand DNA breaks in *C*. *elegans*

**Fig 1:**
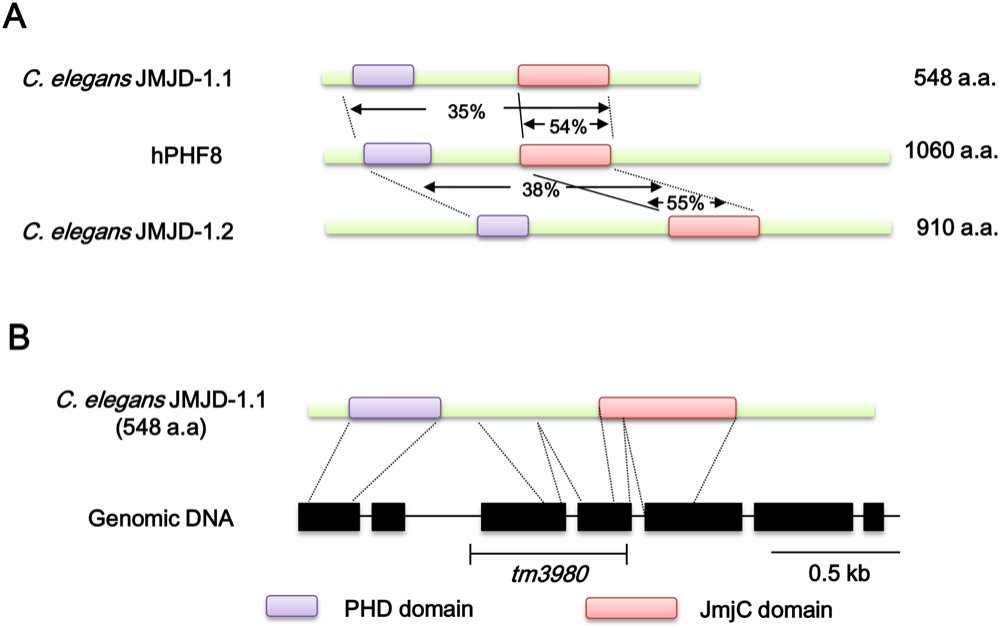
Schematic representation of human PHF8 together with its *C*. *elegans* homologs, and a simplified gene structure of *C*. *elegans jmjd-1*.*1*. (A) Comparison of the amino acid sequences of human histone demethylase PHF8 and its *C*. *elegans* homologs. The alignment of conserved domains and similarity scores were obtained using NCBI Protein Blast. (B) The structure of *C*. *elegans jmjd-1*.*1* (F43G6.6), from exon 1 to 7 (black boxes), and the deletion site in the allele *tm3980*.

JMJD-1.1 and JMJD-1.2 proteins are very similar in amino acid sequences to human PHF8, which is a histone demethylase associated with X-linked mental retardation. There is more than 35% identity between PHF8 and *C*. *elegans* homologs over a 380 amino acid stretch and 55% in the JmjC domain (Fig 1). JMJD-1.1, in particular, has 60% similarity to JMJD-1.2 over the entire length of the polypeptide, suggesting that the two *C*. *elegans* proteins are probably paralogs. We obtained *jmjd-1*.*1(tm3980)* and *jmjd-1*.*2(tm3713)* deletion mutant worms generated by the National Bioresource Project (Japan) and outcrossed them several times with wild-type N2 strain to remove any background mutations. The two mutants have deletions of more than 500 nucleotides containing exons, and are very likely to be null mutants. In the case of *jmjd-1*.*1(tm3980)*, two successive exons are deleted, and the most likely transcript is the second exon joined to the fifth exon. Actually, the transcript was found to encode a prematurely terminated polypeptide of 135 amino acids due to a frameshift, as verified by reverse transcription followed by a gene-specific polymerase chain reaction (S1 Fig). In addition, the level of the transcript in mutant worms amounted to only 20% of that in wild-type N2, as measured by quantitative polymerase chain reaction after reverse transcription (S1 Fig). Therefore, the greatly reduced mRNA level and the premature termination eliminating the JmjC domain suggest that the *jmjd-1*.*1(tm3980)* allele is completely null.

**Fig 2:**
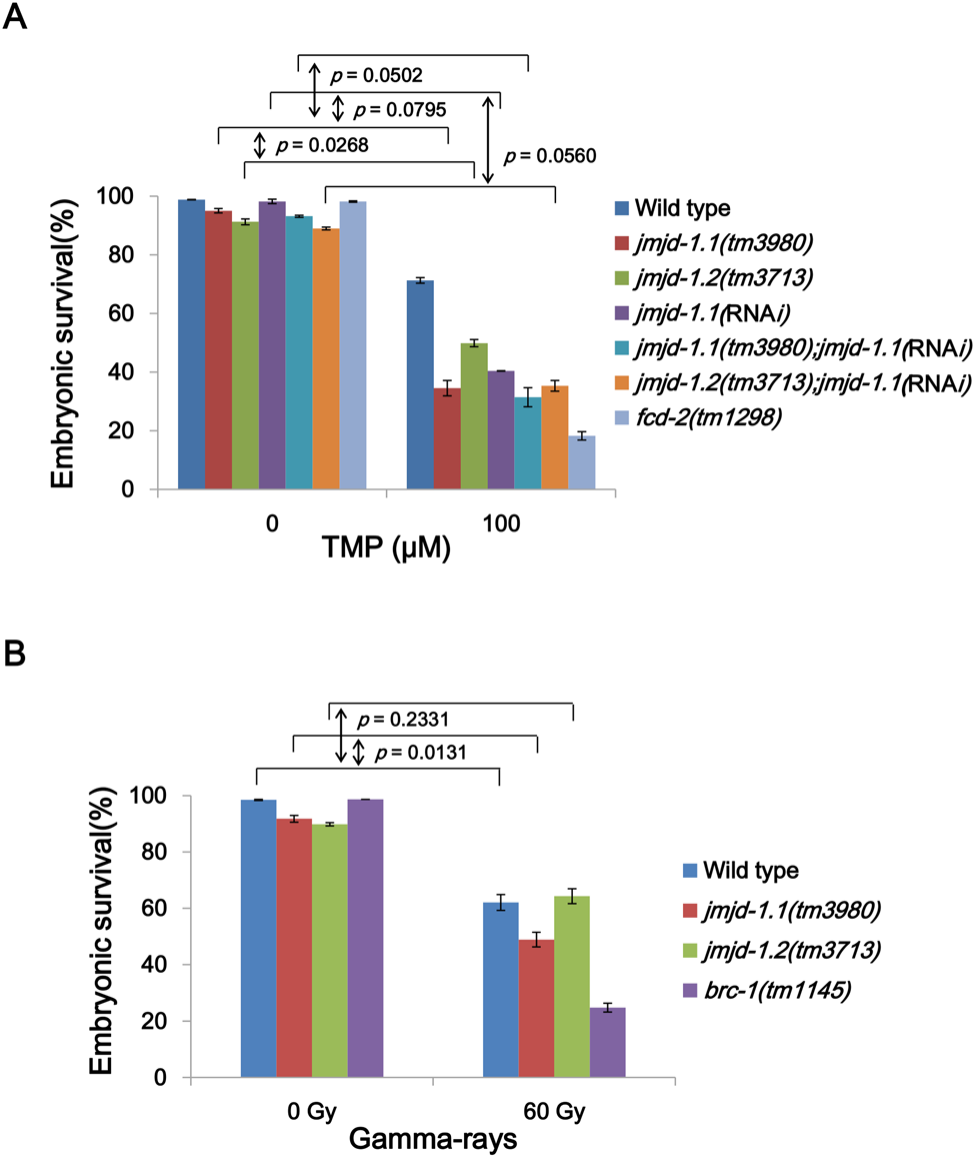
*jmjd-1*.*1* mutant worms are hypersensitive to ICLs and DSBs. (A) L4 stage worms were collected and incubated with trimethyl psoralen (TMP, 100 μM) for 40 min and exposed to UVA light (150 J/m^2^). (B) L4 stage worms were exposed to γ-rays at 60 Gy. In every experiment, eggs were collected between 24 and 40 h post treatment, and their survival was scored 24 h later. Mutations for *fcd-2* and *brc-1* (*C*. *elegans* FANCD2 and BRCA1 homologs, respectively) were used as positive controls for the corresponding type of DNA damage. Error bars indicate SEM. *p* values were obtained by calculating the difference (Δ) in embryonic survival between 0 and 100 TMP for each strain, and comparing the Δ values in the strains by two-way ANOVA.

In order to probe the roles of the histone demethylases in DNA damage responses, the *jmjd-1*.*1(tm3980)* and *jmjd-1*.*2(tm3713)* mutants were subjected to various types of DNA damage at the L4 larval stage (Fig 2). In *C*. *elegans* hermaphrodite gonads, proliferating germ cells undergo promeiotic development to produce oocytes and sperms, and after self-fertilization, early-embryos are laid by the worms. Embryos that developed from treated germ cells in the proliferating region of the gonad were scored for hatching to L1 larvae. Besides the two *jmjd-1* mutants, two other mutants were also used as positive controls for different types of DNA damage, along with wild-type N2 as a negative control. FCD-2 (a *C*. *elegans* FANCD2 homolog) is a key component of the Fanconi anemia (FA) pathway, which participates in the repair of interstrand crosslinks (ICLs) [11]. Therefore, *fcd-2(tm1298*) mutant worms were used as a positive control for hypersensitivity to ICLs. BRC-1 (a *C*. *elegans* BRCA1 homolog) has an indispensable role in promoting end-resection in the initial stage of homologous recombination (HR) [12], and the mutant *brc-1(tm1145)* was used as a positive control for hypersensitivity to DSBs.

When *jmjd-1*.*1(tm3980)* or *jmjd-1*.*2(tm3713)* worms were exposed to ICLs induced by photo-activated psoralen, both mutants were hypersensitive compared to the wild-type (Fig 2A). The *jmjd-1*.*1(tm3980)* mutant showed a higher sensitivity to ICLs than *jmjd-1*.*2(tm3713)* (*p* = 0.0268, two-way ANOVA test), implying a more important role of JMJD-1.1 in repair of ICLs. Since the two mutants were both hypersensitive to ICLs, we tested whether the two corresponding proteins had overlapping functions. For this purpose, we reduced *jmjd-1*.*1* expression by RNA interference (RNA*i*) in wild-type, *jmjd-1*.*1*, and *jmjd-1*.*2* strains. Knockdown of *jmjd-1*.*1* in the wild-type background decreased embryonic survival after ICL treatment as effectively as the *jmjd-1*.*1*(*tm3980)* mutation, indicating almost complete inhibition of JMJD-1.1 expression (*p* = 0.0795, *jmjd-1*.*1(*RNA*i)* vs. *jmjd-1*.*1*(*tm3980)*). As anticipated, knockdown of *jmjd-1*.*1* in *jmjd-1*.*1(tm3980)* worms did not further decrease resistance to ICL treatment (*p* = 0.0502, *jmjd-1*.*1(tm3980);jmjd-1*.*1(*RNA*i)* vs. *jmjd-1*.*1*(*tm3980)*; *p*≥0.05 was treated as not significantly different). This result showed that the knockdown was specific for *jmjd-1*.*1* and also that the hypersensitivity of *jmjd-1*.*1(tm3980)* worms to ICLs did not result from cryptic background mutations. The double deficiency strain *jmjd-1*.*2(tm3713);jmjd-1*.*1(*RNA*i)* was not significantly different from *jmjd-1*.*1(*RNA*i)* in embryonic survival (*p* = 0.0560, *jmjd-1*.*2(tm3713);jmjd-1*.*1(*RNA*i)* vs. *jmjd-1*.*1(*RNA*i)*), pointing to some functional overlap between JMJD-1.1 and JMJD-1.2. A double deficiency strain was also generated by crossing the two single mutants. However, unexpectedly the double mutant was less sensitive to ICLs than either of the single mutant (S2 Fig), unlike the doubly-deficient strains generated by combining one mutation with knockdown of the other gene (*p* = 0.0058, *jmjd-1*.*1(tm3980)* vs. *jmjd-1*.*1(tm3980);jmjd-1*.*2(tm3713)*; *p* = 0.0333, *jmjd-1*.*2(tm3713)* vs. *jmjd-1*.*1(tm3980);jmjd-1*.*2(tm3713)*). A change that compensates for the absence of the two enzyme activities may have occurred in the double mutant during its maintenance over multiple generations.

Only *jmjd-1*.*1(tm3980)* worms showed some hypersensitivity to double-strand DNA breaks (DSBs) induced by γ-rays (*p* = 0.0131, wild-type N2 vs. *jmjd-1*.*1(tm3980)*; *p* = 0.2331, N2 vs. *jmjd-1*.*2(tm3713)* at 60 Gy) (Fig 2B). In addition, embryonic survival after UVC treatment was not affected in either of the mutants (data not shown). Since *jmjd-1*.*1* mutant was more sensitive to ICLs than *jmjd-1*.*2*, and only it was hypersensitive to γ-rays, we focused on the role of JMJD-1.1 in response to DNA damage, especially toward ICLs, in further work.

### JMJD-1.1 is a downstream effector of the Fanconi anemia pathway during ICL repair

**Fig 3:**
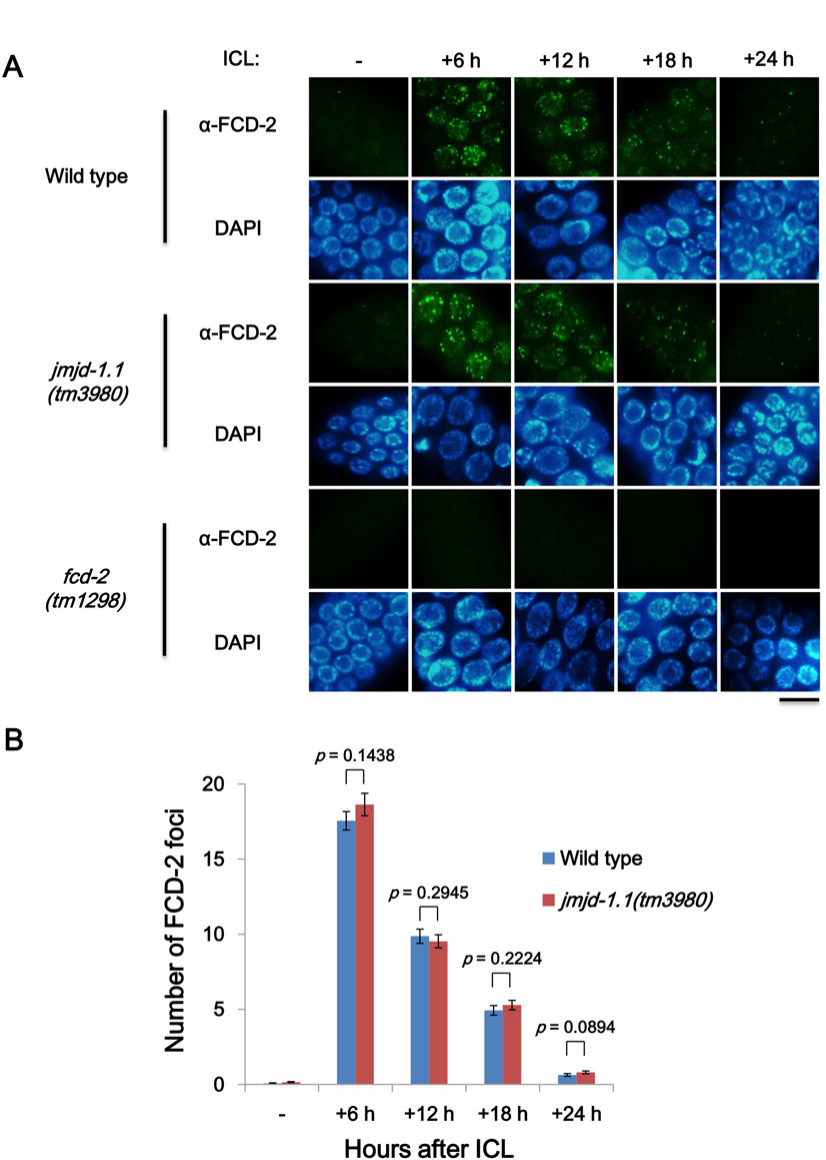
FCD-2 focus formation is unaffected upon ICL induction in mitotic germ cells of *jmjd-1*.*1* worms. L4 stage worms of wild type, *jmjd-1*.*1(tm3980)*, and *fcd-2(tm1298)* were exposed to photoactivatable trimethyl psoralen (TMP, 200 μM) for 40 min and then to UVA light (150 J/m^2^). (A) Gonads were dissected, fixed, and immuno-stained with antibody against FCD-2 (*C*. *elegans* FANCD2 homolog) at 6, 12, 18 and 24 h post treatment. FCD-2 foci were observed in germ cells in the mitotically proliferating region of the gonad, and were not present in the negative control strain, *fcd-2(tm1298)*. Scale bar, 10 μm. (B) The focal plane with the maximum number of FCD-2 foci was chosen for each nucleus of the germ cells, and the numbers of FCD-2 foci per focal plane were compared in wild-type and *jmjd-1*.*1* worms at various time points (n = 100 for each bar). *p* values were obtained by Student’s *t*-test.

In mammalian cells, the FA pathway participates in the initial stage of ICL repair, which involves the recognition of ICLs and their conversion to DSBs. FANCD2 is a key player in the FA pathway, shunting the DSB intermediate to the HR pathway [13–15]. The accumulation of nuclear foci of FCD-2, a FANCD2 homolog in *C*. *elegans*, at ICL sites is a good indicator of successful activation of the FA pathway [16]. We found that the extent of FCD-2 focus formation after ICL treatment in the proliferating mitotic germ cells of *jmjd-1*.*1(tm3980)* and wild-type worms was not significantly different (Fig 3, *p*>0.09 at all the time points, Student’s *t*-test). In addition, the immuno-signal declined gradually to similar extents in the two strains until 24 h. These results indicate that JMJD-1.1 does not affect FCD-2 activation and has a role downstream of the FA pathway in dealing with ICLs.

### Prominent role of JMJD-1.1 in homologous recombination during ICL and DSB repairs

**Fig 4:**
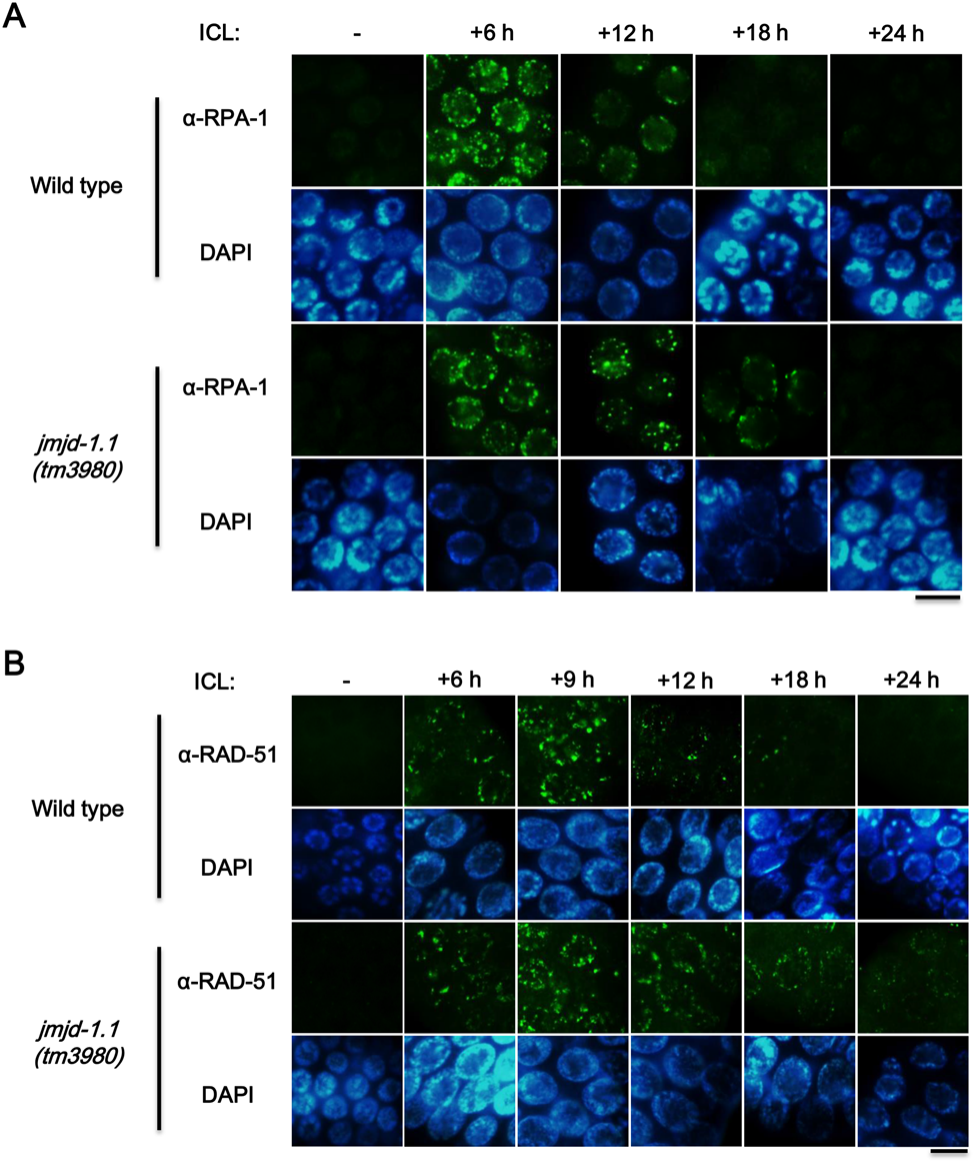
Slow dissipation of RPA-1 and RAD-51 foci induced by ICLs in mitotic germ cells of *jmjd-1*.*1* mutant worms. Prolonged accumulations of (A) RPA-1 (*C*. *elegans* RPA70 homolog) and (B) RAD-51 (*C*. *elegans* RAD51 homolog) foci upon ICL formation in the nuclei of mitotic germ cells of *jmjd-1*.*1* worms. L4 stage worms were exposed to trimethyl psoralen as in Fig 3, and gonads were immuno-stained with the indicated antibodies at 6, 12, 18 and 24 h post treatment (with an additional time point at 9 h in (B)). Scale bar, 10 μm.

ICLs are converted to DSBs in the FA pathway as a result of DNA cleavages by endonucleases such as XPF, MUS81, SLX1, and FAN1 [17,18]. In order for DSBs to be repaired by HR, extensive end resection of DSBs needs to take place; then RPA binds to the exposed single-strand DNA, and RAD51 replaces RPA prior to DNA strand invasion [14]. Mammalian cells defective in HR exhibit prolonged accumulation (or slow dissociation) of RPA and RAD51 proteins at DNA lesions [19,20]. Therefore, we investigated the accumulation and dissociation kinetics of RPA-1 and RAD-51 (*C*. *elegans* RPA70 and RAD51 homologs, respectively) in mitotic germ cells of wild-type and *jmjd-1*.*1(tm3980)* worms. Although these proteins accumulated normally at 6 h after ICL treatment (also at 9 h for RAD-51), striking defects in HR were revealed by the subsequent sluggish dissociation of these proteins in the mutant worms (Fig 4): accumulation of RPA-1 and RAD-51 were still observed in the mutant worms at 18 h post treatment, but they disappeared almost completely in wild-type worms. Similar results were observed for the repair of DSBs induced by γ-rays (S3 Fig). The formation of RPA-1 and RAD-51 foci in mitotic germ cells was normal in the mutant, but their dissociation was slowed down (S3 Fig). In summary, JMJD-1.1 is needed for repair of both ICLs and DSBs via HR, specifically in the step following DSB end resection and subsequent RPA-1/RAD-51 loading.

### The putative histone demethylase JMJD-1.1 modulates heterochromatin structure

**Fig 5:**
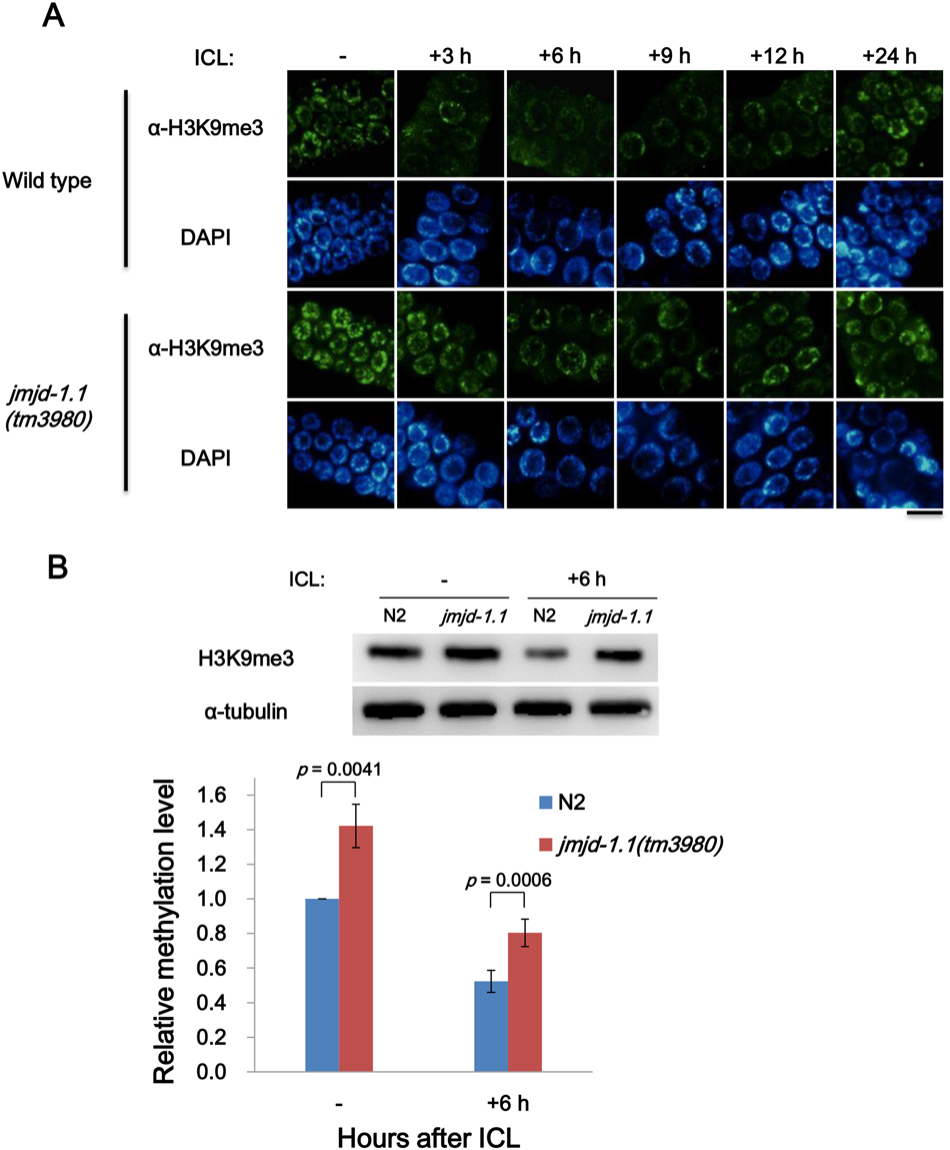
Retarded relaxation of heterochromatin structure upon ICL formation in mitotic germ cells of *jmjd-1*.*1* mutant worms. L4 stage worms were collected and treated with trimethyl psoralen as in Fig 3. (A) The gonads were immuno-stained with antibody against histone H3K9me3 as an indicator of heterochromatin at 3, 6, 9, 12 and 24 h post treatment. Scale bar, 10 μm. (B) After DNA damage induction, worms were grown for a further 6 h, and extracts were prepared. Proteins were separated on a 12% SDS-polyacrylamide gel and transferred to a nitrocellulose membrane. After probing the membrane for histone H3K9me3 and α-tubulin, band intensities were measured and plotted in the bar graph. Each bar represents an average of 9 measurements, obtained in 3 independent experiments each with 3 technical repetitions of the gel electrophoresis. *p* values were obtained by Student’s *t*-test.

Spatiotemporal regulation of chromatin structure is critical for the DNA repair machinery to perform its work efficiently [21,22]. We therefore examined the level of histone H3 tri-methyl Lys9 (H3K9me3), a well-established heterochromatin marker, in mitotic germ cells after ICL or DSB formation. The immuno-signal for H3K9me3 became weaker after DNA damage induction, but it recovered gradually with the progression of DNA repair in both wild-type and mutant cells (Fig 5A and S4 Fig). However, the amount of heterochromatin in the mitotic germ cells of mutant worms was higher than in wild-type worms both before and after the induction of ICLs and DSBs. The differences in H3K9me3 level were also evident in a western analysis of worm extracts (Fig 5B), where the signal for H3K9me3 in the *jmjd-1*.*1* mutant was about 1.5 fold that in the wild type both before and after ICL treatment. The higher level of heterochromatin in the mutant is consistent with the kinetics of RPA-1 and RAD-51 dissociation from DNA lesions in Fig 4 and S3 Fig.

Wild-type worms nearly completed repair of DNA damage within 12 h, as can be seen from the fact that most of RPA-1/RAD-51 had dissociated from the DNA lesions by 12 h post treatment. In contrast, DNA damage in the mutant worms was still under repair for up to 18 h post treatment, as shown by the presence of by non-detached RPA-1/RAD-51. The higher level of heterochromatin, both before and after DNA damage, is likely to be one of the main factors inhibiting DNA repair in the mutant worms. Based on a previous study of the dual demethylase activity of JMJD-1.2 on H3K9me2 and H3K27me2 [9], we carried out immuno-staining to assess any changes in the levels of these two histone signatures in mitotic germ cells. Contrary to our expectation, neither differed between wild-type and *jmjd-1*.*1* worms (S5 Fig; data not shown for H3K27me2), suggesting that JMJD-1.1 targets other signatures such as H3K9me3.

### JMJD-1.1 affects the same step in the HR pathway as RAD-54

**Fig 6:**
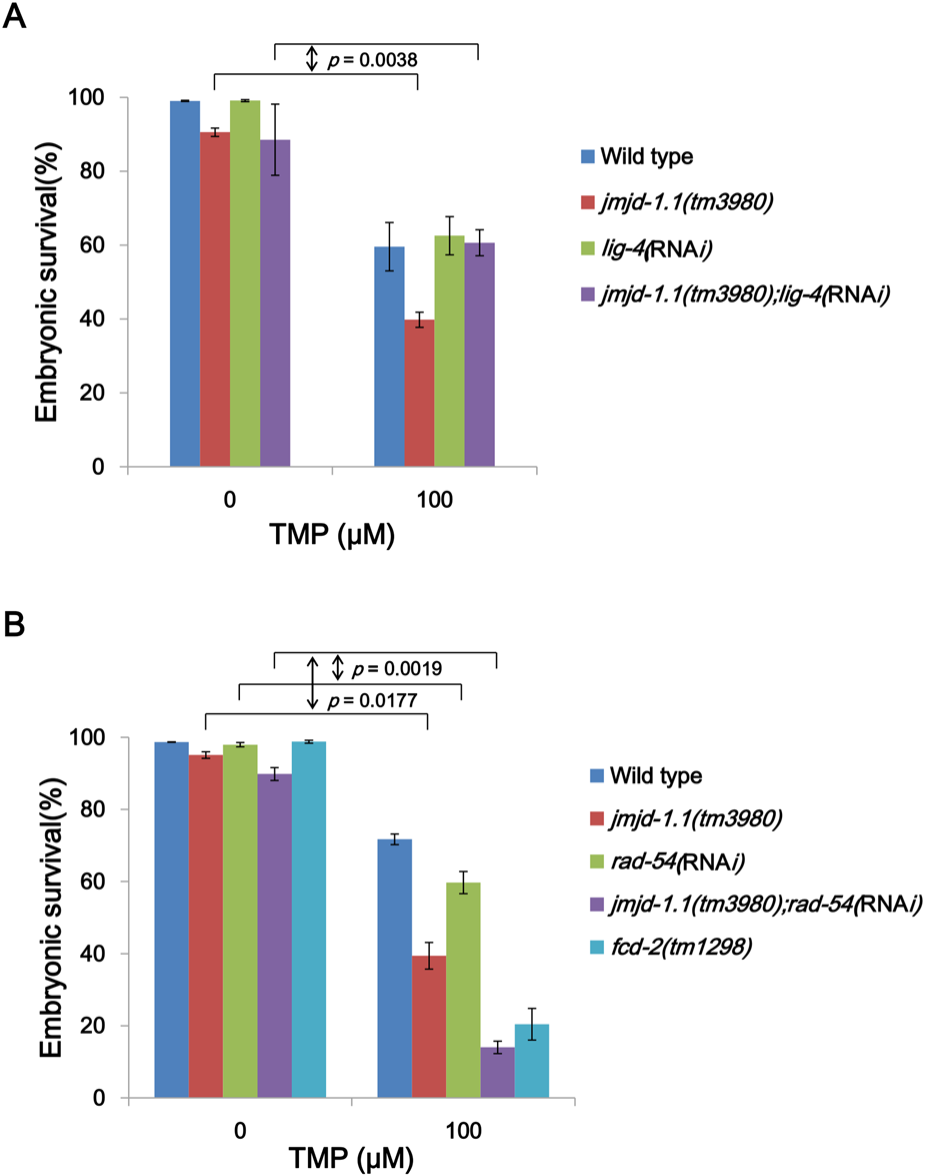
Epistasis test demonstrating a role of JMJD-1.1 in HR, in parallel with RAD-54, in response to ICLs. (A) Wild-type and *jmjd-1*.*1(tm39809)* worms were fed with *E*. *coli* cells expressing double-strand RNA for (A) *lig-4* and (B) *rad-54* from the L1 stage. At the L4 stage, worms were subjected to trimethyl psoralen as in Fig 2. Eggs were collected between 24 and 40 h post treatment, and their survival was scored after 24 h. *p* values were obtained by calculating the differences (Δ) in embryonic survival between 0 and 100 TMP for each strain and comparing the Δ values between strains by two-way ANOVA.

We further addressed the epistatic relationship between JMJD-1.1 and genes of the two main DSB repair pathways, HR and NHEJ (nonhomologous end-joining). For this analysis, we depleted *C*. *elegans rad-54* or *lig-4* (*C*. *elegans* RAD54 or LIG4 homologs, respectively). RAD54 and LIG4 participate in HR and NHEJ, respectively, in mammalian cells, and their conservation in *C*. *elegans* provides excellent ways of turning on and off each of these DSB repair pathways. In several reports, hypersensitivities to DNA damage caused by the absence of key HR factors were reversed by depletion or mutation of crucial components of NHEJ [13,23]. Thus in mammalian systems, phenotypes due to *BRCA1* mutation are rescued by mutation of *53BP1* which inhibits the end-resection of HR and promotes NHEJ [24,25]. Likewise in *C*. *elegans*, the hypersensitivity of *BRCA1* worms to DSBs was rescued by a *LIG4* mutation, and that of *RAD54* knockdown worms was reversed by *53BP1* or *LIG4* mutations [26]. Similarly, the hypersensitivity of *FANCD2* worms and Fanconi anemia patient cells to ICLs was rescued by inhibiting NHEJ [13]. Therefore, we examined the effect of depleting either *lig-4* or *rad-54* in *jmjd-1*.*1* mutants. Since *jmjd-1*.*1(tm3980)* worms are impaired in HR, we expected that the embryonic lethality caused by ICLs might be reversed by depleting *lig-4*. Moreover, if the main role of JMJD-1.1 were in HR, *rad-54* depletion in the mutant background might not have any additional effects on overall embryonic viability. As expected, embryonic lethality due to the *jmjd-1*.*1* mutation was fully rescued by *lig-4* knockdown, supporting the view that JMJD-1.1 is involved in HR during ICL repair (Fig 6A, *p* = 0.0038, *jmjd-1*.*1*(*tm3980);lig-4*(RNA*i)* vs. *jmjd-1*.*1*(*tm3980)*). However, *rad-54* depletion in the *jmjd-1*.*1* mutant background led to massive lethality: death induced by ICLs was 27±2% in the wild type, 56±5% in *jmjd-1*.*1*(*tm3980)*, 38±4% in *rad-54*(RNA*i)*, and 76±4% in *jmjd-1*.*1*(*tm3980);rad-54*(RNA*i)*. This suggested that JMJD-1.1 plays a role in parallel to RAD-54 in HR (Fig 6B, *p* = 0.0177, *jmjd-1*.*1*(*tm3980);rad-54*(RNA*i)* vs. *jmjd-1*.*1*(*tm3980)*; *p* = 0.0019, *jmjd-1*.*1*(*tm3980);rad-54*(RNA*i)* vs. *rad-54*(RNA*i)*). One possible explanation may be that JMJD-1.1 and RAD-54 act at the same step of HR, but have different effects on chromatin structure. In agreement with this view, we have found that knockdown of *rad-54* or use of a hypomorphic mutation of *rad-54* slows down the disappearance of RAD-51 foci after γ-irradiation (data not shown), as does the *jmjd-1*.*1* mutation.

## Discussion

A recent report describing the similarities between human histone demethylase PHF8 and its *C*. *elegans* homolog JMJD-1.2, and the physiological importance of these enzymes led us to examine the roles of the JmjC domain-containing histone demethylases in response to DNA damage [9]. *C*. *elegans* has two homologs of human PHF8, the JmjC domain-containing proteins JMJD-1.1 and JMJD-1.2. PHF8 and the two *C*. *elegans* homologs share two conserved domains: the PHD-finger recognizes and selects target substrate specific for histone demethylase, while JmjC is the catalytic domain (Fig 1) [9]. In this study, we have linked the putative histone demethylase JMJD-1.1 to DNA damage responses, and this in turn led to understanding its activity in terms of chromatin structure. *jmjd-1*.*1(tm3980)* worms exhibit hypersensitivity to ICLs and DSBs (Fig 2), but not to pyrimidine dimers induced by UVC radiation (data not shown). The exceptionally severe effect on embryonic survival after ICL treatment demonstrated that JMJD-1.1 is dedicated more to ICL repair than to DSB repair. However, interestingly, *jmjd-1*.*2(tm3713)* worms showed a milder degree of hypersensitivity to ICLs than *jmjd-1*.*1(tm3980)* worms and no hypersensitivity to DSBs (Fig 2).

The *jmjd-1*.*1* mutation did not affect FCD-2 (FANCD2 homolog) focus formation in response to ICLs, indicating that the initial FA pathway converting ICLs to DSBs was not affected by the mutation. Therefore, the HR pathway acting subsequently to FA pathway was suspected to be defective in the mutant. Indeed, the disappearance of RAD-51 foci not their accumulation was retarded (Fig 4B). The slow disappearance of RAD-51 foci was also observed in *jmjd-1*.*1* after γ-ray treatment, reflecting defects in HR progression (S3 Fig). In addition to RAD-51 foci, RPA-1 foci, which usually form at earlier times than those of RAD-51, also disappeared slowly in the mutant. Thus, JMJD-1.1 appears to function in HR during ICL and DSB repair, at a step after RAD-51 loading onto single-strand DNA. In addition to JMJD-1.1 and JMJD-1.2, a few *C*. *elegans* histone demethylases have been reported to be important for genome stability previously. A H3K4 demethylase SPR-5 is required for normal meiotic homologous recombination and for resistance to double-strand DNA breaks [27,28]. JMJD-2 demethylates H3K9 and H3K36, and its depletion leads to increased apoptosis and RAD-51 focus formation in pachytene-stage germ cells [29].

Immunostaining of *C*. *elegans* germ cells against the heterochromatin signature, histone H3K9me3, strongly supported the notion that the difference in the dissociation kinetics of RPA-1 and RAD-51 between wild-type and *jmjd-1*.*1* worms derived from different chromatin relaxation levels (Fig 5): the immuno-signal was slightly but significantly more intense in *jmjd-1*.*1* germ cells both before and after treatment. This observation shows that JMJD-1.1 acts downstream of the extensive DSB end resection step by modulating chromatin structure.

Nevertheless, accompanying changes in the transcriptome, possibly involving transcripts of DNA repair proteins, may have also contributed to the ICL-hypersensitivity of the *jmjd-1*.*1* mutant.

Because of the retarded relaxation of heterochromatin structure in *jmjd-1*.*1* cells, our next question was which histone signature is modulated by JMJD-1.1. Since JMJD-1.2 has enzymatic activity on histone H3K9me2 and H3K27me2, we thought that JMJD-1.1 might also target these substrates. However, we were not able to distinguish the levels of these two signatures between wild-type and *jmjd-1*.*1* worms after ICL formation (S5 Fig, data not shown for H3K27me2). Interestingly, a homolog of PHF8 and JMJD-1, EPE1, is an antisilencing factor in *S*. *pombe*, as it regulates the extent of heterochromatin domains [30]. The protein is recruited to heterochromatin protein 1 (HP1) bound to methylated histone H3K9 and selectively enriched at heterochromatin boundaries [31].

In an epistasis test, the sensitivity of *jmjd-1*.*1* worms to ICLs reverted to the level of wild-type worms after *lig-4* knockdown. This is very similar to the observation that phenotypes of a mutant of *fcd*-2, which promotes HR on DNA intermediates derived from ICLs, was rescued by a *lig-4* mutation [13]. This similarity between *jmjd-1*.*1* and *fcd-2* in the genetic interaction with *lig-4*, agrees well with our argument that JMJD-1.1 functions in HR. This argument was supported by our results showing the effect of JMJD-1.1 on the disappearance of RAD-51 foci after ICL and DSB formations. In contrast to our expectation based on the role of JMJD-1.1 in HR, *rad-54* RNA*i* synergized with the absence of JMJD-1.1 after ICL treatment. This revealed that JMJD-1.1 in its effect on HR is not fully epistatic to RAD-54. One possible explanation could be that both of JMJD-1.1 and RAD-54 regulates HR by chromatin structure modulation. RAD54 is a SNF2/SWI2 family protein with ATPase activity and is thought to be involved in RAD51 loading and its subsequent dissociation, as well as in branch migration in yeasts and mammals [32]. The RAD54 homolog in *C*. *elegans* affects RAD-51 dissociation (data not shown) as does JMJD-1.1, suggesting that both proteins probably regulate the same step of HR but via different mechanisms. However, another possibility is that JMJD-1.1 also influences a minor DSB repair pathway such as single-strand annealing (SSA) and alternative end-joining (Alt-EJ) as well as HR, thereby showing synergism with RAD-54.

Our research has generated new insights into the role of JMJD-1.1, a putative histone demethylase, in terms of DNA damage responses. Its novelty resides in the fact that *C*. *elegans* JMJD-1.1 and JMJD-1.2 are very similar in terms of amino acid sequence, but have both distinct and overlapping roles in DNA damage responses. Another interesting finding is that JMJD-1.1 affects homologous recombination in response to ICLs and DSBs, probably by modulating chromatin structure in parallel with RAD-54. It will be important to determine which histone proteins JMJD-1.1 acts on to relax chromatin structure and also which repair gene transcripts, if any, it regulates.

## Materials and Methods

### Strains

The standard wild-type strain, Bristol N2, was obtained from the *Caenorhabditis* Genetics Center (Minneapolis, MN, USA). The mutants *jmjd-1*.*1*(*tm3980*), *jmjd-1*.*2(tm3713)*, *fcd-2(tm1298)*, and *brc-1(tm1145)* were generated as part of the National Bioresource Project (Japan). The *jmjd-1*.*1*(*tm3980*) and *jmjd-1*.*2(tm3713)* strains were outcrossed with N2 males 7 and 5 times, respectively, to remove background mutations, while the *fcd-2(tm1298)* and *brc-1(tm1145)* strains were outcrossed 6 and 2 times, respectively. The double mutant *jmjd-1*.*1*(*tm3980);jmjd-1*.*2(tm3713)* was generated by crossing the cognate single mutants. *C*. *elegans* strains were maintained at 20°C on nematode growth medium (NGM) plates seeded with *E*. *coli* OP50-I cells.

### Bacteria-mediated RNA*i*

RNA*i* was performed by feeding *C*. *elegans* with *E*. *coli* HT115(DE3) cells expressing double-stranded RNA (dsRNA) for a given target gene. *E*. *coli* transformants expressing dsRNA for *rad-54* (W06D4.6), *lig-4* (C07H6.1), *jmjd-1*.*1* (F43G6.6), or *jmjd-1*.*2* (F29B9.2) gene were obtained from the *C*. *elegans* RNA*i* v1.1 Feeding Library (Open Biosystems). Cells expressing each dsRNA were first streaked on LB plates containing 50 μg/mL ampicillin, and a single colony was grown at 37°C to an OD_600nm_ of 1.5 in LB medium containing 50 μg/mL ampicillin and 50 μg/mL tetracycline. The cells were then seeded onto solidified NGM plates containing 50 μg/mL ampicillin, 50 μg/mL tetracycline and 1 mM isopropyl β-D-1-thiogalactoside (IPTG), and were used the following day. L1 stage worms were placed on the plates and consumed the cells expressing dsRNA.

### Embryonic survival assay

To induce interstrand DNA crosslinks, L4 stage worms were incubated with 100 μM TMP (4,5,8-trimethylpsoralen, Sigma–Aldrich) for 40 min in a dark chamber. The worms were transferred onto NGM plates and exposed to 150 J/m^2^ UVA (UVL-28 EL, UVP). For double-strand break formation, L4 stage worms were irradiated with 60 Gy of γ-rays from a ^137^Cs source (IBL 437C, CIS Biointernational). Eggs were collected between 24 and 40 h after induction of DNA damage, and their viability into larvae was scored after 24 h.

### Antibodies

*C*. *elegans* FCD-2, RPA-1, and RAD-51 primary antibodies had been previously raised in rats [16], and were used at 1:50, 1:100, and 1:100 dilutions, respectively. The anti-histone primary antibodies against H3 tri-methyl Lys9 and di-methyl Lys9 were purchased from Abcam, and used at 1:50 and 1:20 dilutions in immunostaining, respectively.

### Immunostaining

Worms were placed on glass slides covered with 1x PTw, and their gonads were extracted with an ethanol-cleansed blade. They were fixed in 3% paraformaldehyde (0.1 M K_2_HPO_4_, pH 7.2) for 15 min followed by addition of 1 ml 100% methanol (-20°C). They were then washed five times with 1 ml 1x PTw, and left in 200 μl of blocking solution (goat serum mixed with an equal volume of 1x PTw) for 1 h at room temperature. After blocking, the gonads were incubated with the indicated primary antibody at 4°C overnight. They were then washed five times with 1x PTw and incubated with FITC-conjugated goat secondary antibody (Roche, 1:1000 dilution in blocking solution) matching the host (mouse, rat, or rabbit) of the primary antibody at room temperature for 1 h. After three washes with 1x PTw, the gonads were stained with 1 μg/mL DAPI for 15 min followed by 2 washes with 1x PTw. Lastly, anti-fade solution was added and the gonads were observed under a fluorescence microscope (DMR HC, Leica).

### Western analysis

L4 stage worms were treated with 200 μM photoactivated TMP for 40 min followed by 150 J/m^2^ UVA. The worms were grown further for 6 h and boiled in reducing SDS sample buffer. Proteins were separated by 12% SDS-PAGE and transferred onto a nitrocellulose membrane. Rabbit polyclonal antibody against H3K9me3 (1:500 dilution), mouse monoclonal antibody against H3K9me2 (1:500), and anti-α-tubulin monoclonal mouse antibody (1:5,000) were used as primary antibodies, followed by anti-rabbit or anti-mouse HRP antibodies (Santa Cruz Biotechnology) as secondary antibodies. Electrochemical luminescence assays were performed using WESTSAVEUp (AbFRONTIER). Luminescence signals were detected with a LAS-3000 imaging system (Fujifilm), and the band intensities were measured using a Quantity One version 4.6.6 (Bio-Rad).

## Supporting Information

S1 Fig A shorter mRNA is made from the *jmjd-1*.*1* deletion allele, and at a greatly reduced level. Total RNA was isolated from mixed-stage worms of wild type and *jmjd-1*.*1(tm3980)*, and reverse-transcribed. To determine the splicing pattern of *jmjd-1*.*1* gene in the mutant, primers in the first and fifth exons were used in the amplification of a cDNA fragment, which were 5'-CCGGACTTGAGGAATACGAGTACT and 5'-GCCTCCAAAAGCGAGAGAGTC, respectively. The cDNA sequence showed that splicing occurs between the second and the fifth exons in the mutant (Fig 1). Relative amount of the short *jmjd-1*.*1* transcript with respect to the normal transcript in the wild type was measured by real-time PCR using primers in the fifth exon. The primer pairs were 5′-TCAGGCCCACGAGACGTC and 5′-GCCTCCAAAAGCGAGAGAGTC in the 5th exon.
(PDF)

S2 Fig The double mutant *jmjd-1*.*1(tm3980)*;*jmjd-1*.*2tm3713)* is less sensitive to ICLs than either of the cognate single mutants. L4 stage worms were incubated with 100 μM TMP for 40 min and exposed to UVA light (150 J/m^2^). Eggs were collected between 24 and 40 h post treatment, and their survival was scored 24 h later. Error bars indicate SEM. *p* values were obtained by two-way ANOVA.
(PDF)

S3 Fig Defects in homologous recombination upon DSB formation in mitotic germ cells of *jmjd-1*.*1* worms. Prolonged accumulation of (A) RPA-1 and (B) RAD-51 nuclear foci upon DSB formation in mitotic germ cells of *jmjd-1*.*1* worms. In both experiments, L4 stage worms were exposed to γ-rays (ionizing radiation, IR) at 75 Gy. Gonads were isolated, fixed, and immuno-stained with the indicated antibodies at 6, 12, 18 and 24 h post treatment. Scale bars, 10 μm.
(PDF)

S4 Fig Retarded relaxation of heterochromatin structure upon DSB formation in mitotic germ cells of *jmjd-1*.*1* mutant worms. L4 stage worms were collected and treated with γ-rays at 75 Gy. Gonads were isolated, fixed, and immuno-stained with antibody against histone H3K9me3 as an indicator for heterochromatin at 3, 6, 9, 12 and 24 h post treatment. Scale bar, 10 μm.
(PDF)

S5 Fig No significant differences between wild-type and *jmjd-1*.*1* mutant worms in the di-methylation of histone H3 Lys9 upon ICL formation in mitotic germ cells. **(A)** L4 stage worms were treated with photoactivated TMP as in Fig 3. The gonads were immuno-stained with antibody against histone H3K9me2 at 3, 6, 9, 12 and 24 h post treatment. Scale bar, 10 μm. (B) Worm extracts were prepared 6 h after ICL formation and separated on a 12% SDS-polyacrylamide gel. After transfer to a nitrocellulose membrane, proteins were probed for histone H3K9me2 and α-tubulin. Band intensities were measured and plotted in the bar graph. Each bar represents an average of three independent experiments. *p* values were obtained by Student’s *t*-test.
(PDF)

## References

[1] Kooistra SM, Helin K. Molecular mechanisms and potential functions of histone demethylases. Nat Rev Mol Cell Biol. 2012; 13: 297–311. 10.1038/nrm3327 22473470

[2] Li F, Huarte M, Zaratiegui M, Vaughn MW, Shi Y, Martienssen R, et al Lid2 is required for coordinating H3K4 and H3K9 methylation of heterochromatin and euchromatin. Cell 2008; 135: 272–283. 10.1016/j.cell.2008.08.036 18957202 PMC2614271

[3] Mallette FA, Mattiroli F, Cui G, Young LC, Hendzel MJ, Mer G, et al RNF8- and RNF168-dependent degradation of KDM4A/JMJD2A triggers 53BP1 recruitment to DNA damage sites. EMBO J. 2012; 31: 1865–1878. 10.1038/emboj.2012.47 22373579 PMC3343333

[4] Wakeman TP, Wang Q, Feng J, Wang XF. Bat3 facilitates H3K79 dimethylation by DOT1L and promotes DNA damage-induced 53BP1 foci at G1/G2 cell-cycle phases. EMBO J. 2012; 31: 2169–2181. 10.1038/emboj.2012.50 22373577 PMC3343460

[5] Watanabe S, Watanabe K, Akimov V, Bartkova J, Blagoev B, Lukas J, et al JMJD1C demethylates MDC1 to regulate the RNF8 and BRCA1- mediated chromatin response to DNA breaks. Nat Struct Mol Biol. 2013; 20: 1425–1433. 10.1038/nsmb.2702 24240613

[6] Huang J, Sengupta R, Espejo AB, Lee MG, Dorsey JA, Richter M, et al p53 is regulated by the lysine demethylase LSD1. Nature 2007; 449: 105–108. 17805299 10.1038/nature06092

[7] Pedersen MT, Helin K. Histone demethylases in development and disease. Trends Cell Biol. 2010; 20: 662–671. 10.1016/j.tcb.2010.08.011 20863703

[8] Hoffmann I, Roatsch M, Schmitt ML, Carlino L, Pippel M, Sippl W, et al The role of histone demethylases in cancer therapy. Mol Oncol. 2012; 6: 683–703. 10.1016/j.molonc.2012.07.004 22902149 PMC5528348

[9] Kleine-Kohlbrecher D, Christensen J, Vandamme J, Abarrategui I, Bak M, Tommerup N, et al A functional link between the histone demethylase PHF8 and the transcription factor ZNF711 in X-linked mental retardation. Mol Cell 2010; 38: 165–178. 10.1016/j.molcel.2010.03.002 20346720 PMC2989439

[10] Lin H, Wang Y, Wang Y, Tian F, Pu P, Yu Y, et al Coordinated regulation of active and repressive histone methylations by a dual-specificity histone demethylase ceKDM7A from *Caenorhabditis elegans* . Cell Res. 2010; 20: 899–907. 10.1038/cr.2010.84 20567262

[11] Lee KY, Yang I, Park JE, Baek OR, Chung KY, Koo HS. Developmental stage- and DNA damage-specific functions of *C*. *elegans* FANCD2. Biochem Biophys Res Commun. 2007; 352: 479–485. 17126808 10.1016/j.bbrc.2006.11.039

[12] Boulton SJ, Martin JS, Polanowska J, Hill DE, Gartner A, Vidal M. BRCA1/BARD1 orthologs required for DNA repair in *Caenorhabditis elegans* . Curr Biol. 2004; 14: 33–39. 14711411 10.1016/j.cub.2003.11.029

[13] Adamo A, Collis SJ, Adelman CA, Silva N, Horejsi Z, Ward JD, et al Preventing nonhomologous end joining suppresses DNA repair defects of Fanconi anemia. Mol Cell 2010; 39: 25–35. 10.1016/j.molcel.2010.06.026 20598602

[14] Ciccia A, Elledge SJ. The DNA damage response: making it safe to play with knives. Mol Cell 2010; 40: 179–204. 10.1016/j.molcel.2010.09.019 20965415 PMC2988877

[15] Deans AJ, West SC. DNA interstrand crosslink repair and cancer. Nat Rev Cancer 2011; 11: 467–480. 10.1038/nrc3088 21701511 PMC3560328

[16] Lee KY, Chung KY, Koo HS. The involvement of FANCM, FANCI, and checkpoint proteins in the interstrand DNA crosslink repair pathway is conserved in *C*. *elegans* . DNA Repair 2010; 9: 374–382. 10.1016/j.dnarep.2009.12.018 20075016

[17] Stoepker C, Hain K, Schuster B, Hilhorst-Hofstee Y, Rooimans MA, Steltenpool J, et al SLX4, a coordinator of structure-specific endonucleases, is mutated in a new Fanconi anemia subtype. Nat Genet. 2011; 43:138–141. 10.1038/ng.751 21240277

[18] Kottemann MC, Smogorzewska A. Fanconi anaemia and the repair of Watson and Crick DNA crosslinks. Nature 2013; 493: 356–363. 10.1038/nature11863 23325218 PMC3700363

[19] Godthelp BC, Artwert F, Joenje H, Zdzienicka MZ. Impaired DNA damage-induced nuclear Rad51 foci formation uniquely characterizes Fanconi anemia group D1. Oncogene 2002; 21: 5002–5005. 12118380 10.1038/sj.onc.1205656

[20] Long DT, Räschle M, Joukov V, Walter JC. Mechanism of RAD51-dependent DNA interstrand cross-link repair. Science 2011; 333: 84–87. 10.1126/science.1204258 21719678 PMC4068331

[21] Chiolo I, Minoda A, Colmenares SU, Polyzos A, Costes SV, Karpen GH. Double-strand breaks in heterochromatin move outside of a dynamic HP1a domain to complete recombinational repair. Cell 2011; 144: 732–744. 10.1016/j.cell.2011.02.012 21353298 PMC3417143

[22] Gospodinov A, Herceg Z. Chromatin structure in double strand break repair. DNA Repair 2013; 12: 800–810. 10.1016/j.dnarep.2013.07.006 23919923

[23] Pace P, Mosedale G, Hodskinson MR, Rosado IV, Sivasubramaniam M, Patel KJ. Ku70 corrupts DNA repair in the absence of the Fanconi anemia pathway. Science 2010; 329: 219–223. 10.1126/science.1192277 20538911

[24] Cao L, Xu X, Bunting SF, Liu J, Wang RH, Cao LL, et al A selective requirement for 53BP1 in the biological response to genomic instability induced by Brca1 deficiency. Mol Cell 2009; 35: 234–241. 10.1016/j.molcel.2009.06.037 PMC3392030 19716796

[25] Bunting SF, Callén E, Wong N, Chen HT, Polato F, Gunn A, et al 53BP1 inhibits homologous recombination in Brca1-deficient cells by blocking resection of DNA breaks. Cell 2010; 141: 243–254. 10.1016/j.cell.2010.03.012 20362325 PMC2857570

[26] Ryu JS, Kang SJ, Koo HS. The 53BP1 homolog in *C*. *elegans* influences DNA repair and promotes apoptosis in response to ionizing radiation. PLoS One 2013; 8: e64028 10.1371/journal.pone.0064028 23667696 PMC3648578

[27] Katz DJ, Edwards TM, Reinke V, Kelly WG. A *C*. *elegans* LSD1 demethylase contributes to germline immortality by reprogramming epigenetic memory. Cell 2009; 137: 308–320. 10.1016/j.cell.2009.02.015 19379696 PMC2672044

[28] Nottke AC, Beese-Sims SE, Pantalena LF, Reinke V, Shi Y, Colaiácovo MP. SPR-5 is a histone H3K4 demethylase with a role in meiotic double-strand break repair. Proc Natl Acad Sci USA 2011; 108: 12805–12810. 10.1073/pnas.1102298108 21768382 PMC3150895

[29] Whetstine JR, Nottke A, Lan F, Huarte M, Smolikov S, Chen Z, et al Reversal of histone lysine trimethylation by the JMJD2 family of histone demethylases. Cell 2006; 125: 467–481. 16603238 10.1016/j.cell.2006.03.028

[30] Ayoub N, Noma K, Isaac S, Kahan T, Grewal SI, Cohen A. A novel jmjC domain protein modulates heterochromatization in fission yeast. Mol Cell Biol. 2003; 23: 4356–4370. 12773576 10.1128/MCB.23.12.4356-4370.2003 PMC156127

[31] Braun S, Garcia JF, Rowley M, Rougemaille M, Shankar S, Madhani HD. The Cul4-Ddb1^Cdt2^ ubiquitin ligase inhibits invasion of a boundary-associated antisilencing factor into heterochromatin. Cell 2011; 144: 41–54. 10.1016/j.cell.2010.11.051 21215368 PMC3645473

[32] Agarwal S, van Cappellen WA, Guénolé A, Eppink B, Linsen SE, Meijering E, et al ATP-dependent and independent functions of Rad54 in genome maintenance. J Cell Biol. 2011; 192: 735–750. 10.1083/jcb.201011025 21357745 PMC3051825

