# Supplementary figures and images for: A PHF8 Homolog in C. elegans Promotes DNA Repair via Homologous Recombination
Source: PLoS One. 2015 Apr 8;10(4):e0123865. doi: 10.1371/journal.pone.0123865 (PMC4390335; doi:10.1371/journal.pone.0123865)

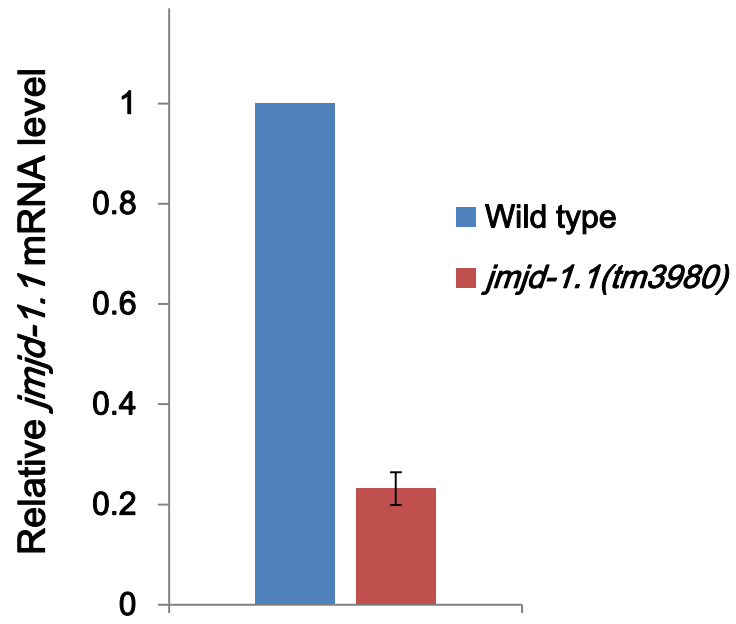

Supplement: S1 Fig — Total RNA was isolated from mixed-stage worms of wild type and jmjd-1.1(tm3980), and reverse-transcribed. To determine the splicing pattern of jmjd-1.1 gene in the mutant, primers in the first and fifth exons were used in the amplification of a cDNA fragment, which were 5'-CCGGACTTGAGGAATACGAGTACT and 5'-GCCTCCAAAAGCGAGAGAGTC, respectively. The cDNA sequence showed that splicing occurs between the second and the fifth exons in the mutant (Fig 1). Relative amount of the short jmjd-1.1 transcript with respect to the normal transcript in the wild type was measured by real-time PCR using primers in the fifth exon. The primer pairs were 5′-TCAGGCCCACGAGACGTC and 5′-GCCTCCAAAAGCGAGAGAGTC in the 5th exon. (PDF) [file pone.0123865.s001.pdf]

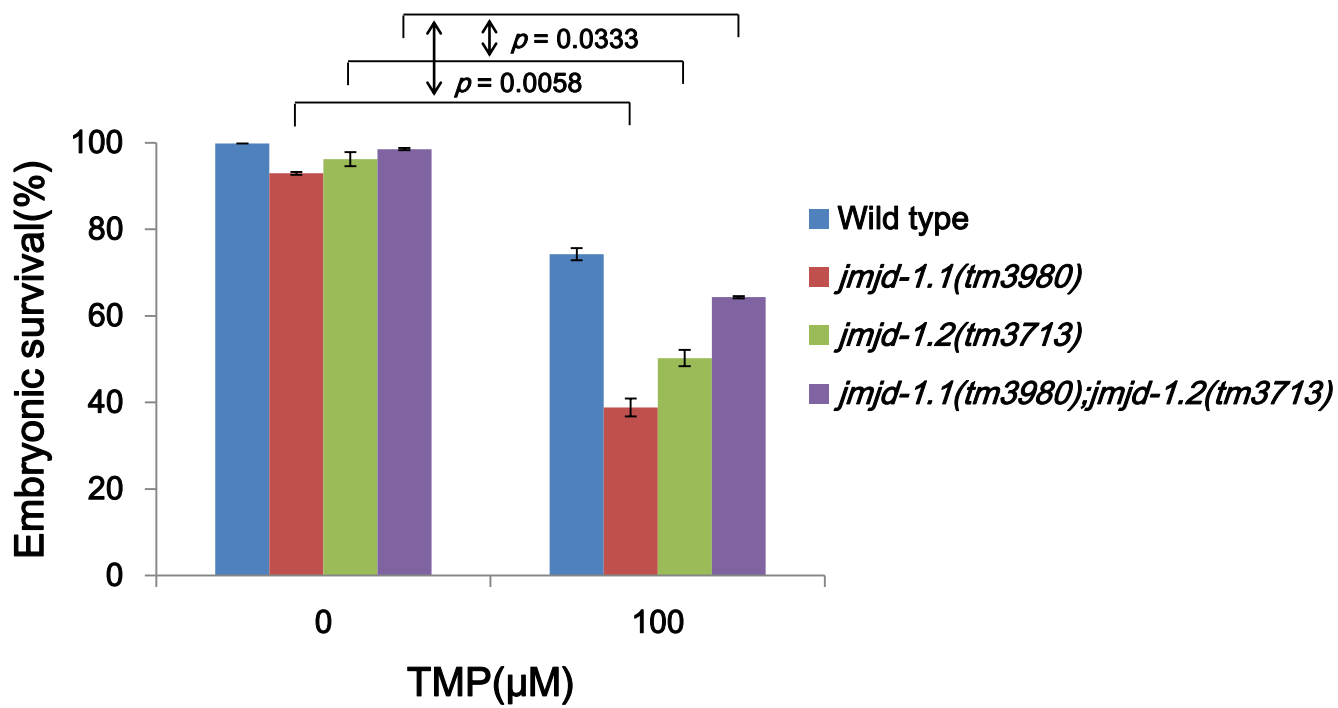

Supplement: S2 Fig — L4 stage worms were incubated with 100 μM TMP for 40 min and exposed to UVA light (150 J/m2). Eggs were collected between 24 and 40 h post treatment, and their survival was scored 24 h later. Error bars indicate SEM. p values were obtained by two-way ANOVA. (PDF) [file pone.0123865.s002.pdf]

**A**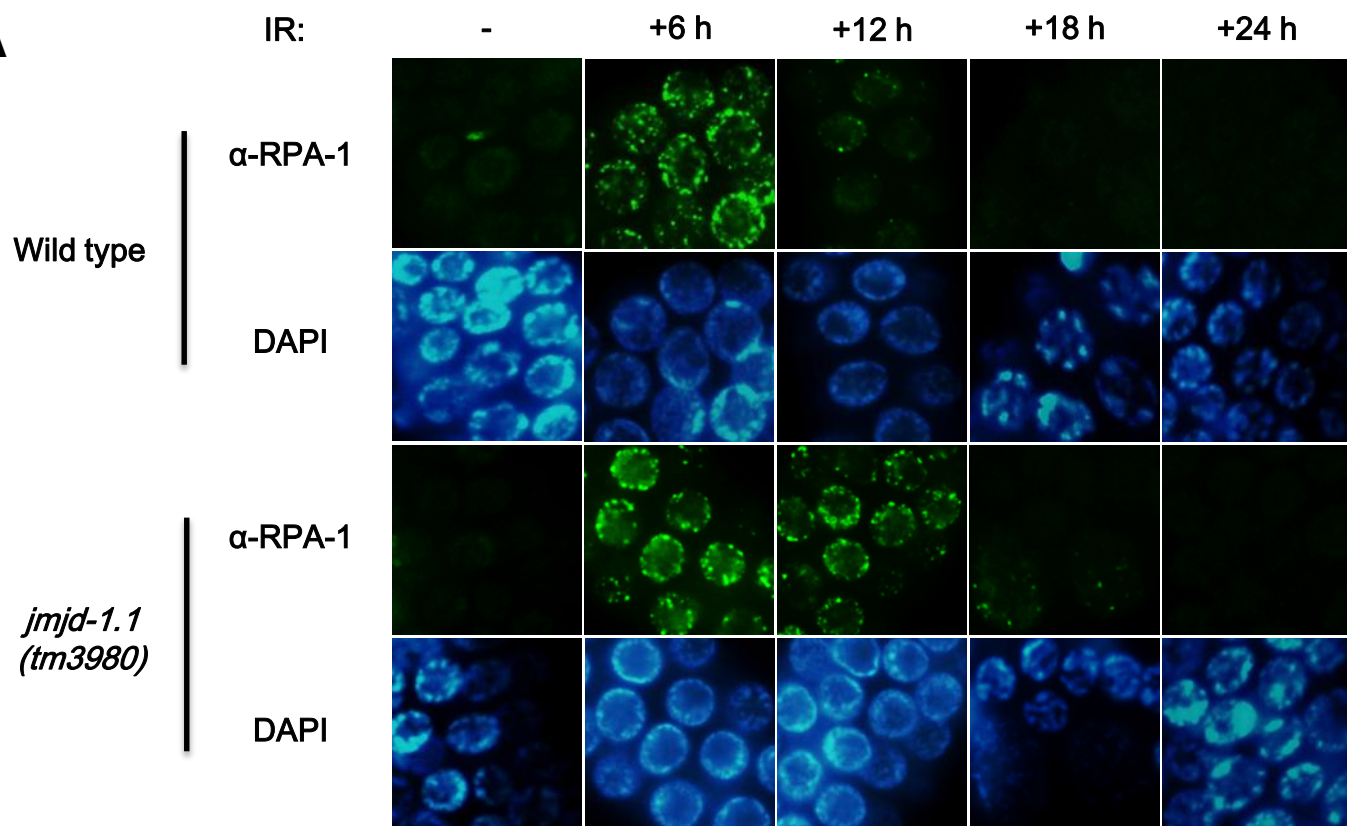**B**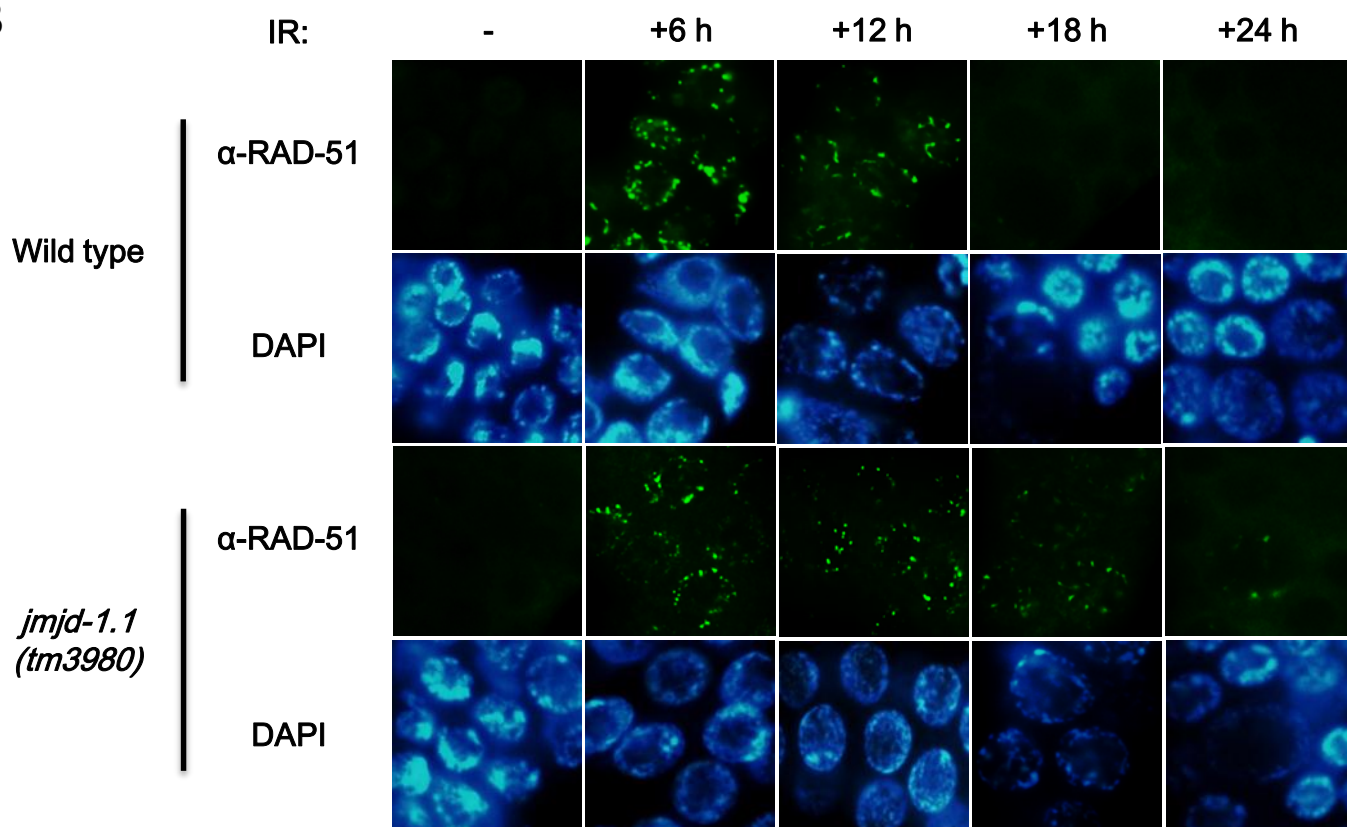

Supplement: S3 Fig — Prolonged accumulation of (A) RPA-1 and (B) RAD-51 nuclear foci upon DSB formation in mitotic germ cells of jmjd-1.1 worms. In both experiments, L4 stage worms were exposed to γ-rays (ionizing radiation, IR) at 75 Gy. Gonads were isolated, fixed, and immuno-stained with the indicated antibodies at 6, 12, 18 and 24 h post treatment. Scale bars, 10 μm. (PDF) [file pone.0123865.s003.pdf]

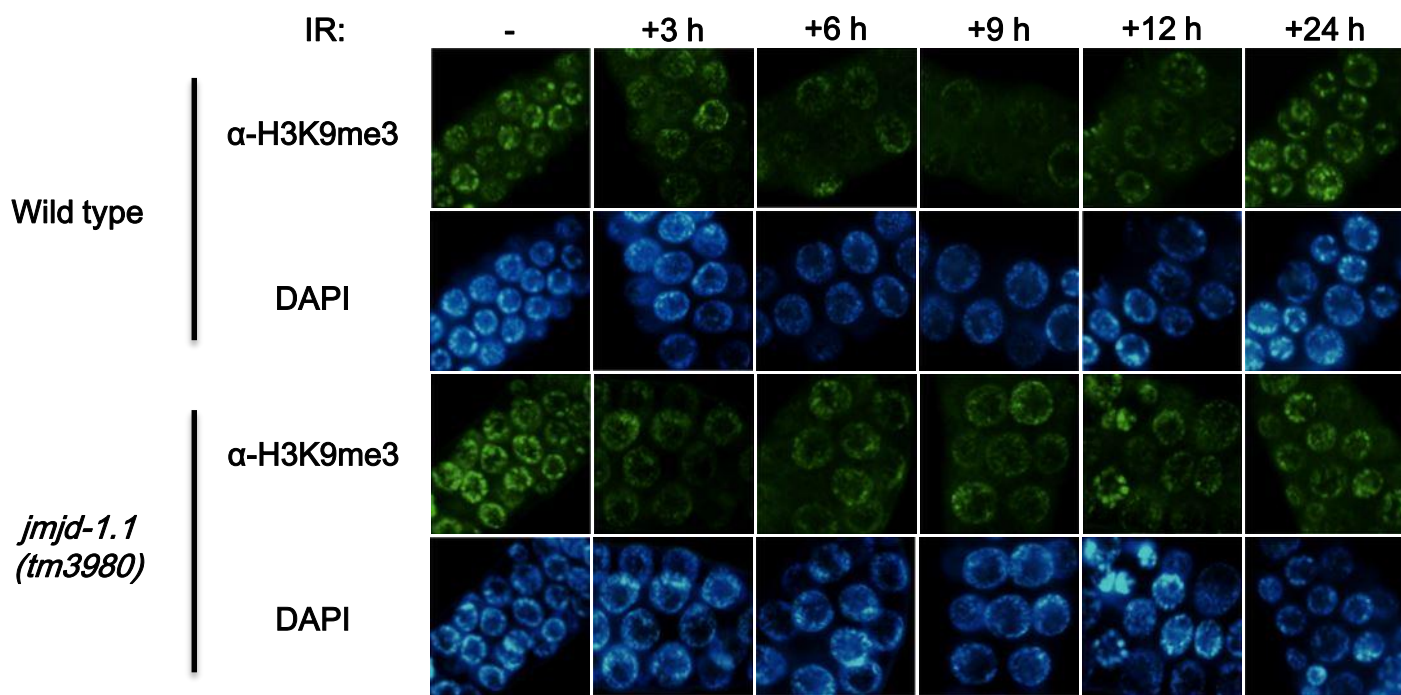

Supplement: S4 Fig — L4 stage worms were collected and treated with γ-rays at 75 Gy. Gonads were isolated, fixed, and immuno-stained with antibody against histone H3K9me3 as an indicator for heterochromatin at 3, 6, 9, 12 and 24 h post treatment. Scale bar, 10 μm. (PDF) [file pone.0123865.s004.pdf]

**A**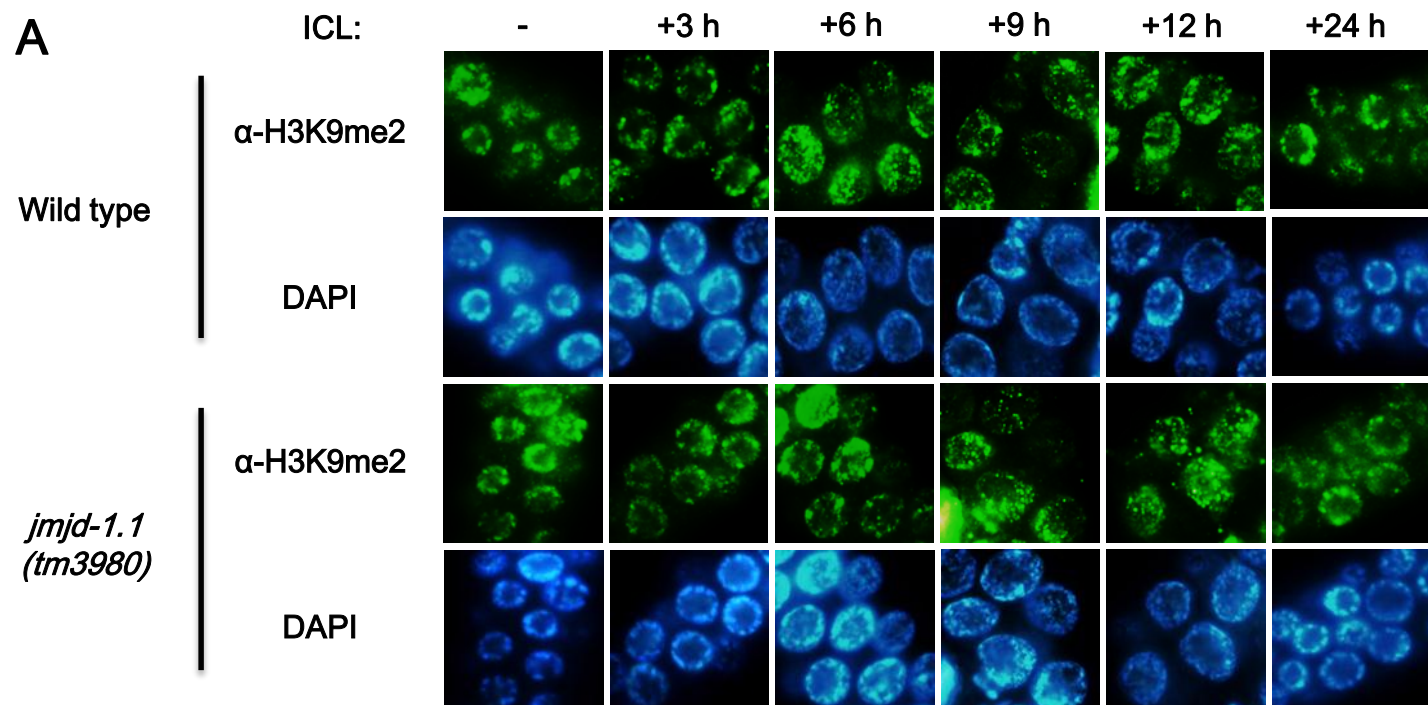**B**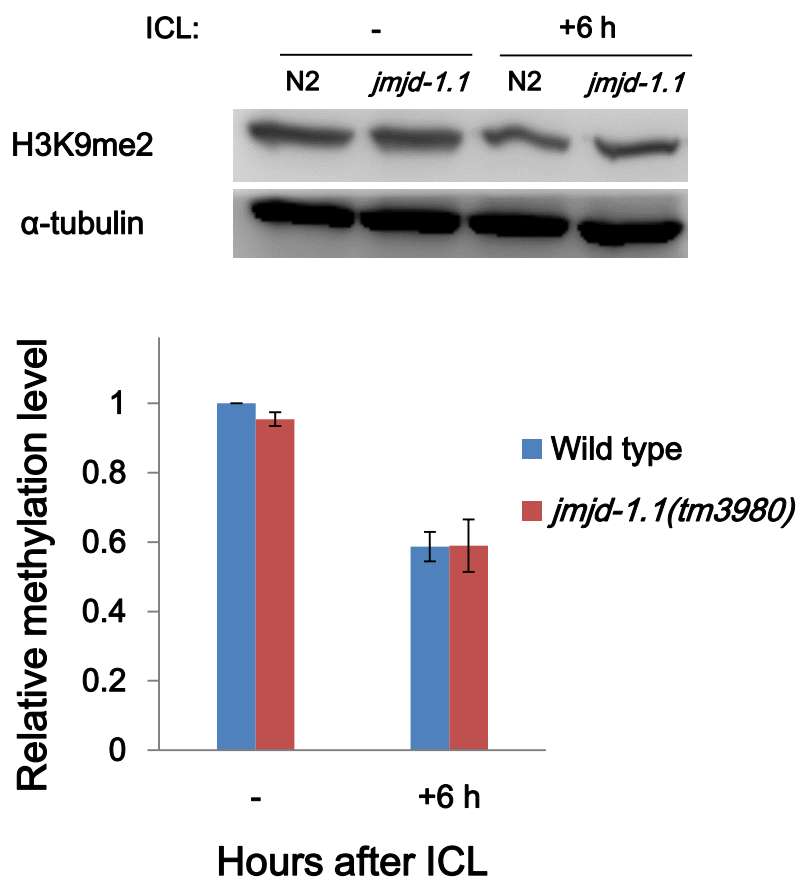

Supplement: S5 Fig — (A) L4 stage worms were treated with photoactivated TMP as in Fig 3. The gonads were immuno-stained with antibody against histone H3K9me2 at 3, 6, 9, 12 and 24 h post treatment. Scale bar, 10 μm. (B) Worm extracts were prepared 6 h after ICL formation and separated on a 12% SDS-polyacrylamide gel. After transfer to a nitrocellulose membrane, proteins were probed for histone H3K9me2 and α-tubulin. Band intensities were measured and plotted in the bar graph. Each bar represents an average of three independent experiments. p values were obtained by Student’s t-test. (PDF) [file pone.0123865.s005.pdf]
